# Personalized ventilation guided by electrical impedance tomography with increased PEEP improves ventilation‐perfusion matching in asymmetrical airway closure and contralateral pulmonary embolism during veno‐venous extracorporeal membrane oxygenation: A case report

**DOI:** 10.14814/phy2.70280

**Published:** 2025-04-11

**Authors:** Roberta Garberi, Claudio Ripa, Gianmarco Carenini, Luca Bastia, Marco Giani, Giuseppe Foti, Emanuele Rezoagli

**Affiliations:** ^1^ School of Medicine and Surgery University of Milano‐Bicocca Monza Italy; ^2^ Anesthesia and Intensive Care Unit AUSL Romagna, M. Bufalini Hospital Cesena Italy; ^3^ Department of Anesthesia and Intensive Care Fondazione IRCCS San Gerardo dei Tintori Monza Italy

**Keywords:** acute respiratory distress syndrome (ARDS), asymmetrical airway closure, case report, electrical impedance tomography (EIT), pulmonary embolism (PE)

## Abstract

We report the case of a 54‐year‐old man with right‐lung pneumonia and contralateral pulmonary embolism (PE) conditioning severe refractory hypoxemia requiring veno‐venous extracorporeal membrane oxygenation. Electrical impedance tomography (EIT) was used to assess recruitability and regional ventilation and perfusion. At a clinical positive‐end expiratory pressure (PEEP) of 12 cmH₂O, EIT revealed predominant ventilation in the left lung and predominant perfusion in the right lung. Reduced perfusion in the left lung raised suspicion of PE, confirmed by contrast‐enhanced computed tomography. The clinical PEEP was insufficient to maintain recruitment of the pneumonia‐affected right lung, which showed an airway opening pressure (AOP) of 16 cmH₂O. Therefore, PEEP was increased to 20 cmH₂O to exceed the AOP of the injured lung, improving lung recruitment, stabilizing end expiratory lung impedance (EELI), and increasing V/Q matching. Oxygenation improved, following an increased cardiac output, and reduced pulmonary vascular resistance. Despite increasing ventilation pressures, the higher PEEP was well‐tolerated hemodynamically, optimizing V/Q coupling in this case of unilateral shunt and contralateral dead space. This case highlights the utility of ventilation/perfusion EIT in optimizing ventilatory strategies, in anticipating the presence of pulmonary malperfusion at bedside, and demonstrating the importance of individualized, physiology‐based interventions in complex critical care scenarios.

## 1 INTRODUCTION

Asymmetrical lung injury presents a complex and challenging scenario in the management of mechanically ventilated patients. Positive end‐expiratory pressure (PEEP) can facilitate alveolar recruitment in the injured lung, but it carries the risk of shifting tidal volume and end‐expiratory lung volume toward the healthier lung (Amigoni et al., 2013). This redistribution may lead to overdistension and increase the likelihood of ventilator‐induced lung injury (VILI).

The presence of contralateral pulmonary embolism (PE) further complicates the clinical scenario. By redirecting perfusion to the non‐embolized lung, PE exacerbates ventilation‐perfusion (V/Q) mismatch, creating a delicate balance between achieving adequate oxygenation and avoiding hemodynamic compromise. Addressing these dual challenges requires a careful and individualized approach to mechanical ventilation.

Electrical impedance tomography (EIT) provides a dynamic, bedside tool to evaluate the distribution of ventilation and perfusion in real time. While traditionally used to monitor ventilation patterns, EIT has recently gained traction for assessing regional pulmonary perfusion in critically ill patients (Spinelli et al., 2021). Its ability to provide simultaneous analyses of ventilation and perfusion dynamics makes it particularly valuable in managing complex cases.

## 2 CASE REPORT

A 54‐year‐old man, a former smoker with a history of hypertension, presented to the emergency department with worsening dyspnea following a 3‐day history of fever and cough. A computed tomography (CT) scan confirmed predominant right‐lung pneumonia as the underlying condition. On the first day of admission, the patient underwent a trial of noninvasive ventilation which failed to achieve adequate oxygenation. This necessitated endotracheal intubation and the initiation of invasive mechanical ventilation.

Despite maximal ventilatory support, his respiratory status continued to decline, leading to veno‐venous extracorporeal membrane oxygenation (VV‐ECMO) on the third day of hospitalization to manage severe refractory hypoxemia. Even with full ECMO support (Blood Flow (BF) 4 L/min, FiO_2_ 100% both at the natural lung (NL) and the membrane lung (ML), Gas Flow (GF) 3 L/min), the patient remained significantly hypoxic, with a PaO_2_ of 59 mmHg.

Given the asymmetrical lung injury, electrical impedance tomography (EIT) was performed to assess lung recruitability and evaluate regional ventilation and perfusion.

The present case report was prepared in accordance with the CARE checklist.

The patient was deeply sedated and paralyzed, ventilated in pressure‐regulated volume‐controlled (PRVC) mode with a tidal volume (Vt) of 480 mL and a set respiratory rate (RR) of 10 breaths per minute. He was positioned in a semi‐recumbent position with the head of the bed elevated to 30°.

EIT was performed using a 16‐electrode belt (PulmoVista 500, Dräger, Lübeck, Germany) placed between the 4th and 5th intercostal spaces. The mattress was flattened, and pulsation therapy was discontinued in order to avoid interference with EIT measurements. Data were acquired at a sampling frequency of 50 Hz and saved for offline analysis.

Regional pulmonary perfusion was evaluated using the bolus technique, based on the first‐pass kinetics of a 10 mL bolus of 5% hypertonic solution. Data analysis was conducted with EIT Perfusion Analysis V1.2.0 software (Dräger, Lübeck, Germany) (Borges et al., 2012).

**FIGURE 1:**
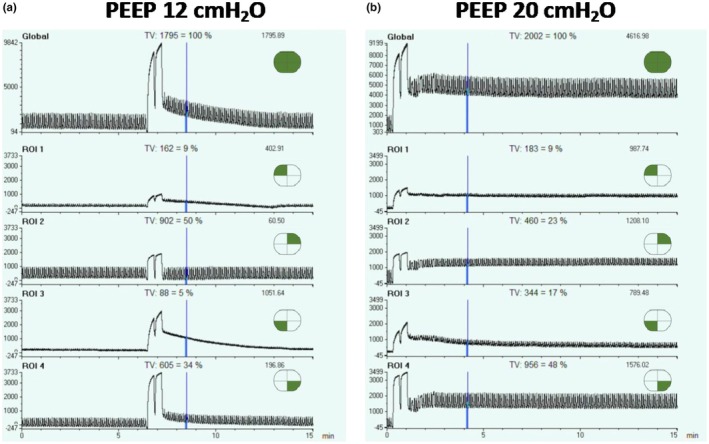
Panels (a) and (b) illustrate electrical impedance tomography (EIT) recordings obtained over 15 min following a recruitment maneuver (RM). This RM consisted of two sustained inflations at 40 cmH₂O, each lasting 20 s, performed at PEEP levels of 12 cmH₂O and 20 cmH₂O, respectively. The regions of interest (ROIs) are designated as follows: ROI 1 and ROI 3 represent the ventral and dorsal regions of the right lung, while ROI 2 and ROI 4 correspond to the ventral and dorsal regions of the left lung. In Panel (a), the EIT recordings show an initial rise in end‐expiratory lung impedance (EELI) immediately after the RM, which then rapidly decayed over time. Specifically, the right lung, affected by pneumonia, exhibited a progressive loss of EELI, as indicated by the marked decline in impedance signals over time in ROI 1 (ventral) and ROI 3 (dorsal). Conversely, in the left lung, which was affected by pulmonary embolism, ROI 2 (ventral) showed no significant increase in EELI post‐recruitment, whereas ROI 4 (dorsal) displayed an initial rise in EELI, which gradually decreased over time. The tidal ventilation distribution images further highlight that ventilation was predominantly confined to the left lung. This is evidenced by clear impedance variations in ROI 2 and ROI 4, in contrast to the minimal impedance changes observed in ROI 1 and ROI 3 of the right lung. Panel (b) shows that the increased PEEP raised global EELI, which remained stable over time, though minor decay persisted in the dorsal region of the right lung (ROI 3). Tidal ventilation in ROI 1 and 3 of the right lung increased, as indicated by the greater amplitude of the impedance variation in these ROIs compared to PEEP of 12 cmH_2_O. However, the higher PEEP caused overdistension in ventral regions, as evidenced by reduced impedance variation in ROI 2 of the left lung. Regional compliance analysis further confirmed this pattern: At 12 cmH₂O PEEP, ventral compliance was 27 mL/cmH₂O compared to 21 mL/cmH₂O in dorsal regions; at 20 cmH₂O PEEP, ventral compliance decreased to 15 mL/cmH₂O, while dorsal compliance increased to 29 mL/cmH₂O.

At clinical PEEP of 12 cmH_2_O, a recruitment maneuver (RM) consisting of two insufflations at 40 cmH_2_O for 20 s each was performed. The RM resulted in a transient increase in end‐expiratory lung impedance (EELI), predominantly in the right lung, though this effect decayed over time (Figure 1).

**FIGURE 2:**
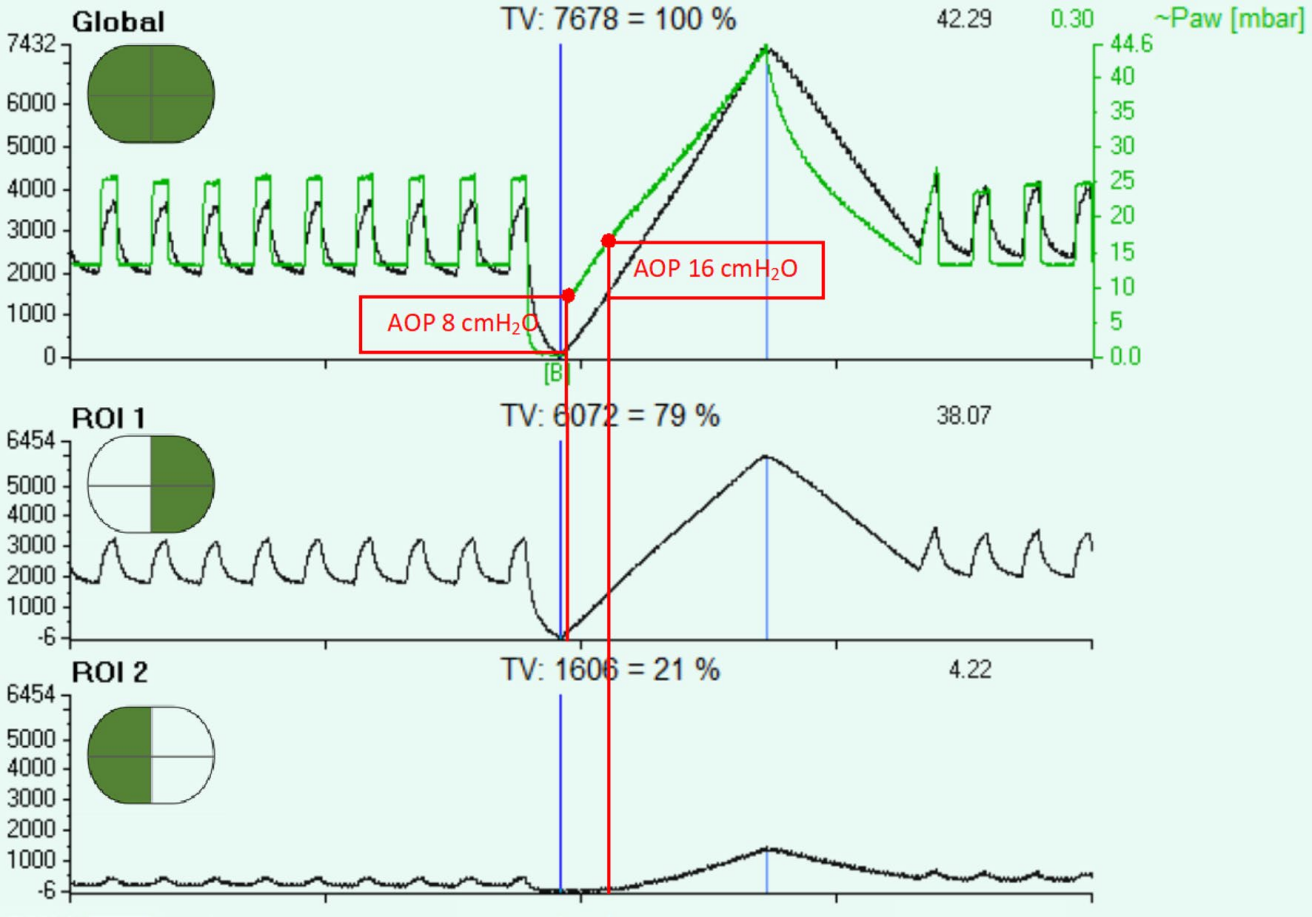
The image shows the EIT recording obtained during a low flow inflation maneuver. It highlights the differential Airway Opening Pressure (AOP) in the right and left lungs (Rozé et al., 2024). Region of Interest 1 (ROI 1) represents the left lung, affected by pulmonary embolism (PE), with an AOP of 8 cmH₂O, while ROI 2 represents the right lung, affected by pneumonia, with an AOP of 16 cmH₂O.

A low‐flow inflation maneuver revealed an airway opening pressure (AOP) of 8 cmH_2_O, which was lower than the set PEEP level (Tisminetzky et al., 2023). When analyzed by lung region, the AOP was 8 cmH₂O in the left (healthier) lung and 16 cmH₂O in the right (injured) lung (Rozé et al., 2021) (Figure 2).

Given the differential AOP and the loss of EELI over time, a second RM was performed, followed by an increase in PEEP to 20 cmH_2_O, targeting a plateau pressure of 28–30 cmH_2_O with protective ventilation (Mercat et al., 2008) to stabilize lung recruitment and prevent further EELI loss.

The increased PEEP raised global EELI, which remained stable over time, though minor decay persisted in the dorsal region of the right lung (ROI 3).

However, the higher PEEP caused overdistension in ventral regions, as evidenced by reduced impedance variation in ROI 2 of the left lung.

**FIGURE 3:**
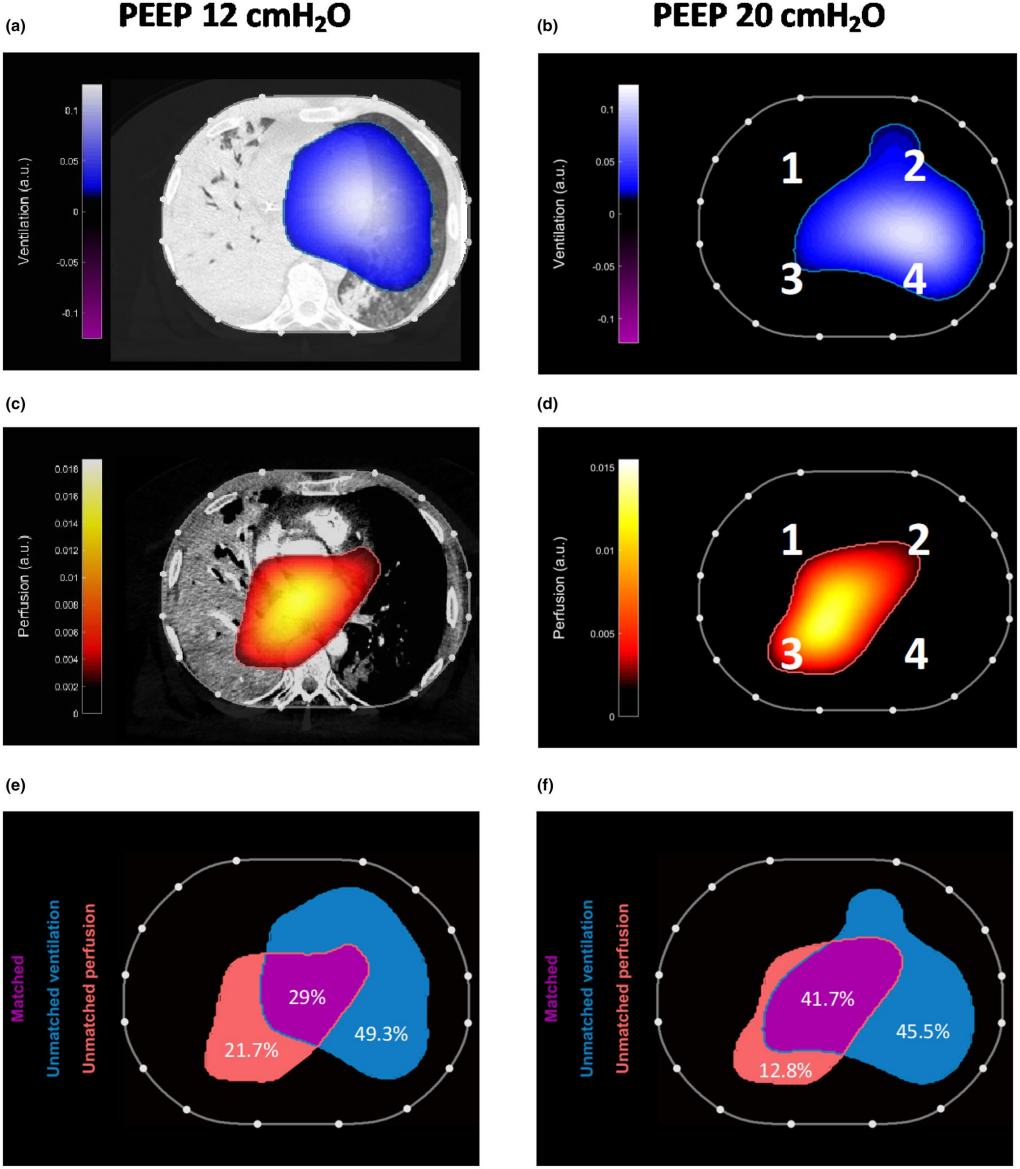
Panels (a) and (b) display ventilation at PEEP 12 and 20 cmH_2_O, respectively. Panels (c) and (d) display perfusion at PEEP 12 and 20 cmH_2_O, respectively. Panel (a) also includes a CT scan highlighting the lung parenchyma. The data indicate that ventilation was predominantly directed to the left lung, which was unaffected by pneumonia, while perfusion was primarily concentrated in the right lung. This mismatch was attributed to a pulmonary embolism affecting the left lung, a finding subsequently confirmed by a contrast‐enhanced CT scan, shown in Panel (c). Panel (e) illustrates ventilation and perfusion matching at a clinical PEEP of 12 cmH₂O, while Panel (f) demonstrates that increasing the PEEP to 20 cmH₂O improved the extent of matched ventilation‐perfusion regions (from 29% to 41.7%, in purple). This adjustment significantly reduced unmatched perfusion (i.e., shunt, in red) from 21.7% to 12.8%, and decreased unmatched ventilation (i.e., dead space, in blue), from 49.3% to 45.5%.

At both PEEP levels, ventilation predominantly occurred in the left lung, which was marginally affected by pneumonia, while perfusion remained concentrated in the right lung. This uneven perfusion was due to a pulmonary embolism (PE) in the left lung. This EIT finding was later confirmed by a contrast‐enhanced CT scan (Figure 3) and clarified the cause of the patient's persistent hypoxemia: the well‐ventilated left lung was poorly perfused, increasing dead space, while the poorly aerated right lung showed relatively high perfusion, contributing to shunt.

EIT analysis demonstrated that increasing PEEP to 20 cmH₂O improved ventilation‐perfusion matching, reduced unmatched perfusion (dead space) and reduced unmatched ventilation (shunt) (Figure 3). Correspondingly, at PEEP 20 cmH_2_O, the patient's PaO_2_ improved from 59 mmHg to 66 mmHg.

Notably, cardiac output measured by a Swan‐Ganz pulmonary catheter also increased at 20 cmH_2_O, rising from 8.5 L/min at PEEP 12 cmH_2_O to 9.8 L/min, while ECMO blood flow remained unchanged. This indicates that the increase in PaO_2_ was observed even in the presence of a higher cardiac output. Furthermore, SvO_2_ increased from 84% to 85.5%, accordingly.

The increase in PEEP also reduced pulmonary vascular resistance (PVR), which decreased from 169 dyn•s/cm^5^•m^2^ at 12 cmH₂O to 122 dyn•s/cm^5^•m^2^ at 20 cmH₂O.

At PEEP 20 cmH₂O, the wedge pressure increased to 18 mmHg, compared to 12 mmHg at PEEP 12 cmH₂O. Systolic/diastolic pulmonary pressures showed a slight rise with higher PEEP, increasing from 44/30 mmHg to 47/33 mmHg.

Over the following days, the patient showed gradual clinical improvement. The PE was managed with a thromboaspiration procedure scheduled for the day after the EIT study, which successfully restored pulmonary circulation. Following the procedure, FiO_2_ was reduced from 100% to 75% at both the ML and NL and PaO_2_ increased to 102 mmHg, despite an increase in CO to 13 L/min and a decrease in BF to 3.8 L/min.

On the 10th day of ECMO, the patient was successfully weaned. By the 17th day of ICU, he was extubated and subsequently transferred to a general ward on the 19th day of hospitalization to continue recovery. A diagnostic workup identified cryptogenic organizing pneumonia (COP) with a micronodular pattern as the underlying etiology, with no microbiological pathogens isolated during the clinical course. The patient was discharged home in stable condition on the 36th day of hospitalization.

## 3 DISCUSSION

The ventilatory management of asymmetric lung injury with a consequent asymmetrical airway closure is challenging due to the coexistence of lung regions with different mechanical properties, that may make difficult the identification of an optimal ventilatory setting. The situation becomes even more complex when pulmonary embolism (PE) affects the contralateral lung, leading to two distinct ventilation‐perfusion (V/Q) mismatch phenomena: shunt in the pneumonia‐affected lung (Raimondi Cominesi et al., 2024) and dead space in the embolism‐affected lung (Rezoagli et al., 2022).

In this context, using a low positive end‐expiratory pressure (PEEP) may exacerbate shunt through derecruitment in perfused but consolidated lung areas (Bastia et al., 2021). Conversely, a high PEEP may increase dead space by overdistending the PE‐affected lung and elevating right ventricular afterload. This makes EIT and lung perfusion studies especially valuable in such situations by estimating at the same time the potential for recruitment and ventilation‐perfusion matching noninvasively at the bedside, potentially improving protective ventilation strategies (Bastia et al., 2021; Spinelli et al., 2021).

In our case, EIT demonstrated that at low PEEP, ventilation and perfusion were asymmetrical and uncoupled. Ventilation was predominantly directed to the left lung, which was less affected by pneumonia, while perfusion was concentrated in the right lung. Furthermore, the assessment of an amputation of perfusion in the left lung by EIT raised suspicion of a pulmonary embolism. This anticipated diagnosis at the bedside was later confirmed by contrast‐enhanced computed tomography (CT). Thus, the EIT perfusion study was instrumental in identifying the PE and guiding subsequent diagnostic confirmation.

Although the use of high PEEP in asymmetrical lung injury is controversial due to the risk of overdistending the healthier lung, in our case, it resulted in: (1) overall more homogeneous ventilation distribution; (2) stabilized end‐expiratory lung impedance (EELI) across both lungs; and (3) improved V/Q matching.

We believe that higher PEEP levels overcame airway opening pressure in the pneumonia injured lung and likely decreased lung heterogeneity in an asymmetrical lung injury (Rozé et al., 2024). Setting PEEP above the AOP of the injured lung may have helped prevent atelectrauma and improve oxygenation by reducing ventilation/perfusion mismatch (Rozé et al., 2021), as demonstrated by the increased matched regions observed in the EIT study.

Importantly, the increase in PEEP did not compromise the patient's hemodynamics. At a PEEP of 20 cmH₂O, CO improved, likely reflecting enhanced right ventricular function due to reduced pulmonary vascular resistance (PVR). The decrease in PVR may have been driven by different variables such as improved SvO_2_ and a reduced shunt. The reduction in PVR likely resulted from alveolar recruitment, which decreased shunt, and from decreased hypoxic pulmonary vasoconstriction (HPV) in previously collapsed lung regions, thanks to a higher SvO_2_.

These mechanisms likely underpinned the observed improvements in cardiovascular performance. Furthermore, the increased lung resting volume in the contralateral noninjured lung may have promoted some of the cardiac output to be directed majorly to the right injured lung, thereby reducing some of the overall dead space component of the hypoxemia.

Nevertheless, the rise in pulmonary artery wedge pressure indicated increased pulmonary venous pressures, potentially due to elevated intrathoracic pressures from higher PEEP. However, since the heart rate remained constant after the PEEP increase—around 115 beats per minute—the rise in CO was likely due to an increase in stroke volume, suggesting that the wedge pressure increased as a consequence of the higher stroke volume.

In conclusion, the application of higher PEEP led to (1) Improved oxygenation, (2) Increased cardiac output driven by reduced PVR, and (3) Mild increase in pulmonary artery and wedge pressures associated with a higher stroke volume.

This case highlights the utility of EIT for guiding ventilatory management of complex cases of asymmetrical airway closure because of asymmetrical lung injury and for suggesting noninvasively at bedside diagnosis of contralateral pulmonary embolism. Tailoring PEEP based on real‐time regional ventilation and perfusion data improved V/Q matching, oxygenation, and hemodynamics, illustrating the importance of individualized, physiology‐driven strategies in this challenging case.

## FUNDING INFORMATION

Support was provided solely from institutional and/or departmental sources.

## CONFLICT OF INTEREST STATEMENT

The authors declare that they have no conflicts of interest.

## ETHICS STATEMENT

The study was conducted in accordance with the Declaration of Helsinki. It did not require formal approval from the institutional review board, as the interventions were performed according to the standard of care at our institution. The patient provided informed consent for the publication of the case.

## Data Availability

The data analyzed are available from the corresponding author upon reasonable request.
